# Reduced stem nonstructural carbohydrates caused by plant growth retardant had adverse effects on maize yield under low density

**DOI:** 10.3389/fpls.2022.1035254

**Published:** 2022-10-20

**Authors:** Qian Tang, Jianhong Ren, Xiong Du, Shiduo Niu, Shanshan Liu, Dejie Wei, Yarong Zhang, Dahong Bian, Yanhong Cui, Zhen Gao

**Affiliations:** State Key Laboratory of North China Crop Improvement and Regulation/ Key Laboratory of Water-Saving Agriculture in North China, Ministry of Agriculture and Rural Affairs/ Key Laboratory of Crop Growth Regulation of Hebei Province/ College of Agronomy, Hebei Agricultural University, Baoding, China

**Keywords:** plant growth retardant, planting density, carbohydrate, enzyme activity, polyamines, yield components

## Abstract

Enhancing maize lodging resistance with plant growth retardants (PGRs) is common in maize production. However, the underlying mechanisms of yield formation as affected by PGRs are still poorly understood. A field experiment contained PGR application (a mixture of ethephon and cycocel, EC) with normal (T1) and double (T2) doses and water control (CK) was conducted at four maize plant densities (4.5, 6.0, 7.5, and 9.0 plants m^−2^) in 2020 and 2021. In this two-year study, the grain yield and kernel number per ear (KNE) of EC treatments were reduced by 4.8–9.0% and 3.3–12.2%, respectively, compared with CK under densities of 4.5, 6.0, and 7.5 plants m^−2^ without lodging. However, under the density of 9.0 plants m^−2^, EC treatments had no pronounced effects on grain yield and yield components. Across all densities, EC significantly decreased the leaf area index (LAI), and the lowest LAI was recorded in T2. The concentrations of nonstructural carbohydrates (NSCs; starch and soluble sugar) in the stem were significantly decreased by 9.9–10.2% in T2 averaged all densities. The sucrose and starch concentrations in grains also declined in the EC treatments. The key enzymes (cell wall acid invertase, sucrose synthase, and adenosine diphosphate pyrophosphorylase) and grain polyamine concentrations showed a slight downward trend under EC treatments compared to CK. NSCs in stems and grains, kernel enzyme activities, and polyamines in grains presented significant positive correlations with KNE. Additionally, structural carbohydrate (SC; including cellulose, hemicellulose, and lignin) concentrations in stems were improved with enhanced lodging resistance by spraying EC. Significant negative relationships were observed between SC with kernel number m^-2^ (KNM) and yield, suggesting that improved SC in stems might affect the availability of NSCs for kernel set. Although the lowest kernel weight and KNE were obtained at 9.0 plant m^−2^, relatively high LAI still ensured high KNM and high yield. Collectively, EC treatment increased SC in stems, enhanced lodging resistance of maize and reduced NSC availability for kernels, ultimately presenting adverse effects on maize kernel number and yield under relative low density.

## Introduction

Maize (*Zea mays* L.) is the most extensively grown crop, with the highest total grain yield globally (Zhang et al., 2014). The demand for maize grain yield has been increasing with global population growth (Grassini et al., 2011; Feng et al., 2021; Liu et al., 2021). Increasing the planting density, which has been proved to contribute to 8.5–17.0% of maize grain yield, is the most effective way to improve yield (Assefa et al., 2018). However, high density is accompanied by high risk of lodging and kernel abortion (Xue et al., 2017; Tang et al., 2018). Lodging destroys the normal canopy structure, causes decrease in photosynthetic capacity and accumulated photosynthates, and eventually results in yield reduction (Xue et al., 2016; Shu et al., 2019). Spraying plant growth retardants (PGRs) containing ethephon, an alternative measure to enhance the lodging resistance of maize, can increase the yield by reducing the lodging rate (Gong et al., 2021; Huang et al., 2021), while grain yield loss is frequently observed under PGRs when lodging does not occur (Cox and Andrade, 1988; Norberg et al., 1988).

In general, kernel number m^-2^ (KNM) and 1000-kernel weight (TKW) codetermine maize yield (Borrás and Vitantonio-Mazzini, 2018). Both traits vary in maize across environments, such as PGR use, plant density, and adverse external pressure (Cox and Andrade, 1988; Borrás and Gambín, 2010; Ren et al., 2022). However, kernel number variation is responsible for most grain yield fluctuations under numerous constraints (Borrás and Vitantonio-Mazzini, 2018; Gao et al., 2018). The kernel number depends on the floret number and kernel setting rate (Zhao et al., 2021). Total number of completely developed floret of maize is a stable genetic trait and almost unaffected by environmental factors, such as drought and high density (Pagano et al., 2007; Oury et al., 2016a). Previous studies indicated that grain development can be impaired by low cell wall acid invertase activity and restricted assimilate availability through asynchronous pollination time (Otegui and Melon, 1997; Bonelli et al., 2016; Li et al., 2018; Shen et al., 2018). Sucrose injection, a way of enhancing assimilate supply, can improve acid invertase activity and consequently alleviate the diminished kernel number under shade and drought (Boyle et al., 1991; Hiyane et al., 2010; Altuntas et al., 2019). Gao et al. (2020) showed that raised carbohydrates at silking in stem could significantly increase maize kernel number. Moreover, adequate carbohydrates are conducive to polyamine biosynthesis, which was associated with early endosperm development (Liang and Lur, 2002) and kernel number (Tiburcio and Alcazar, 2018; Alcazar et al., 2020). Feng et al. (2011) also found that enhanced polyamine could increase kernel number. However, it is still not clear that the effects of carbohydrates, enzyme activities, and polyamines on kernel number under PGR.

Additionally, although the recommended dose of PGRs significantly enhances lodging resistance, the lodging rate of maize can still reach 20–40% at high density (Xu et al., 2017; Zhang et al., 2019). Increasing the PGR dose can significantly reduce plant height and ear height, further improving the amount of structural carbohydrates (SCs) in first to third internodes above ground, e.g., cellulose, hemicellulose, and lignin (Zhang et al., 2020). Research indicated that changes in the nonstructural carbohydrates (NSCs) and SCs are negatively correlated in sorghum hybrids (McBee and Miller, 1993). However, the relationships between NSC and SC in maize are still unclear. Meanwhile, the sugar contents in lodging resistance and kernel abortion are largely unknown (Shah et al., 2021). Both of NSC and SCs (cellulose, hemicellulose) were important sinks for photosynthates (Sekhon et al., 2016). SCs contributed much to enhancing lodging resistance (Shah et al., 2021). NSCs serve as components of “the buffer system” in the maize source–sink relationship, providing an alternative transient sink for photosynthates and carbohydrates for grain growth (Slewinski, 2012). Therefore, we hypothesized that increased stem mechanical strength by PGRs would increase SC and decrease NSC availability, which had a detrimental effect on kernel set.

A commercial PGR product, “Jindele”, a mixture of ethephon and cycocel (EC), is widely employed to enhance the lodging resistance of maize in China (Zhao et al., 2022). This two-year field experiment with spraying EC at different planting densities was conducted to determine (I) if the balance of NSC and SC in stems is responsible for kernel number and yield and (II) whether NSC, sink activity, and polyamine changes in grains are correlated with kernel number and yield.

## Materials and methods

### Experimental site

**Figure 1:**
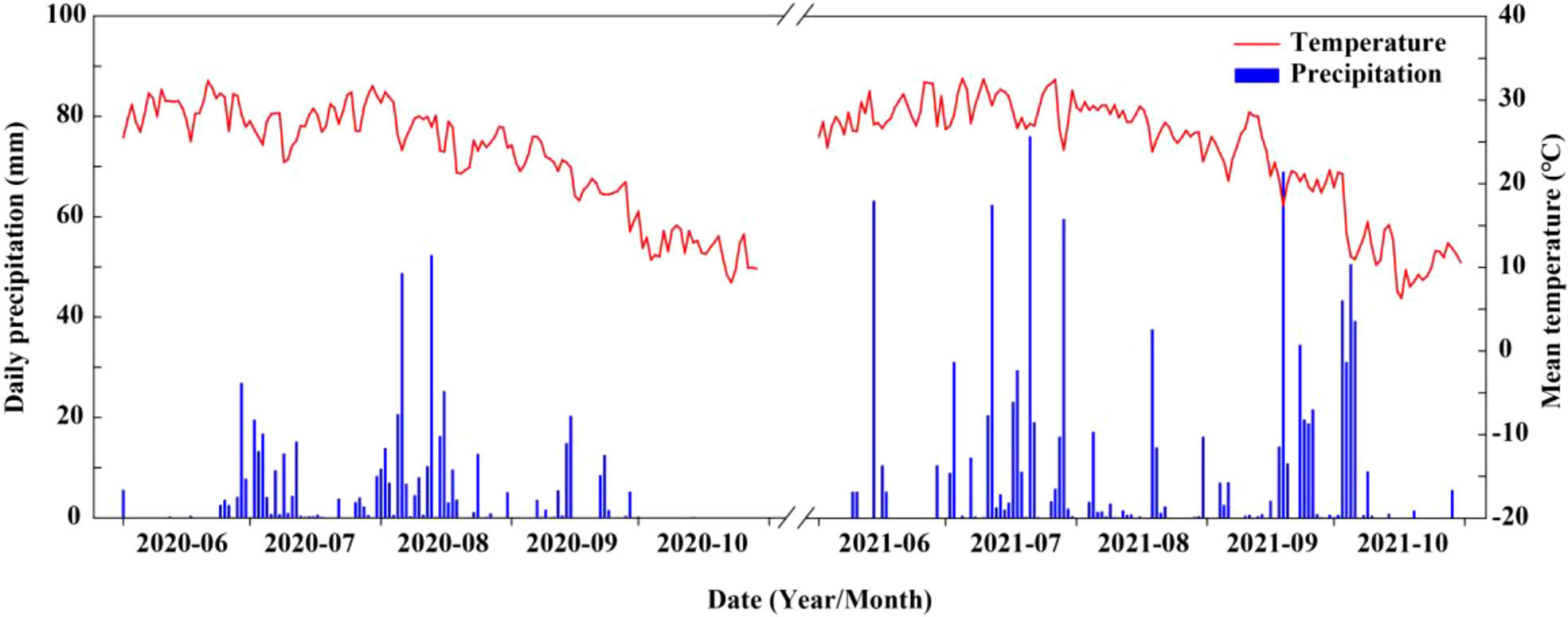
Mean temperature and daily precipitation during maize growing seasons at the experimental site in 2020 and 2021.

The field experiment was carried out at the Shenzhou Dryland Farming Experimental Station of the Hebei Academy of Agriculture and Forestry Sciences (Hebei Province, China, 37° 91′ N, 115° 71′ E) in 2020 and 2021. The soil of the field experiment site was a clay loam containing 12.5 g kg^−1^ total organic matter, 65.8 mg kg^−1^ total nitrogen, 121.9 mg kg^−1^ available potassium, and 15.3 mg kg^−1^ available phosphorus. In addition, daily precipitation and mean temperature for both years are shown in **Figure 1**. Total precipitation during summer maize growth period was 508.2 mm and 736.8 mm in 2020 and 2021, respectively, and average temperature during the same period was 25.4 °C and 26.8 °C, respectively.

### Experimental design and field management

The experiment was conducted in a split-plot design with three replications. The main factor was density (4.5 (D1), 6.0 (D2), 7.5 (D3), and 9.0 (D4) plants m^-2^), and the sub-factor was EC (ethephon: cycocel = 3:1) application (recommended concentration of 2 mL L^−1^ (T1), double concentration of 4 mL L^−1^ (T2), and water control (CK)). At V7 (the 7^th^ leaf was fully expanded), when stem started rapidly elongation, PGR could effectively inhibit stem growth and reduce lodging (Xue et al., 2016). EC (225 L ha^−1^) was uniformly sprayed on the surface of maize leaves in the afternoon (16:00–19:00). The plot area was 80 m^2^ (8 m × 10 m). The widely cultivated maize variety ‘Zhengdan 958’ was used. Manual sowing was carried out on June 13, 2020, and June 15, 2021, with a row spacing of 0.6 m. Before sowing, 750 kg per hectare of compound fertilizer with a ratio of 25:8:12 of N:P_2_O_5_:K_2_O was applied. After sowing, 750 m^3^ of water per hectare was immediately irrigated to ensure emergence. Weeds, plant diseases, and pests were effectively controlled.

### Sampling and measurements

#### Relevant agronomic traits

At the silking stage, three maize plants were randomly selected from each plot to measure agronomic traits. First, plant height, ear height, and center of gravity height were measured (Xu et al., 2017). The stem diameter of the basal first internode above ground was then immediately measured with a vernier caliper.

Afterward, the length and maximum width of each green leaf per maize plant were determined with a ruler. When half or more of leaf was yellowed, it was considered to have senesced. The leaf area index (LAI) was the total leaf area per unit area. The leaf area was calculated using the following formula:

$$Leaf\;area(m^{2})=0.75\;\times \;length\;\times \;width$$

Finally, the lower stem, middle stem (ear internode and the above and below internodes), upper stem (including sheath), leaves, and ear were placed in an oven at 105°C for 30 min and then dried at 80°C to a constant weight. The dry samples were weighed and ground with a grinder and passed through a 1-mm mesh sieve to determine structural and non- structural carbohydrates.

Three maize plants were selected in each plot at 12 days after silking (DAS) in 2021 to count the number of silks per ear (SE) by cutting all silks-out bracts off.

### Stem carbohydrates

Stem NSCs were measured with three independent biological replicates by the anthrone–sulfuric acid method with minor modifications at the silking stage (Hansen and Møller, 1975; Liang et al., 2021). Stem NSCs were extracted in boiling water for 30 min after adding 6 mL of distilled water to 50 mg of the sample. The samples were cooled to room temperature and centrifuged at 1900 *g* for 15 min. The above operation was repeated twice. The supernatant was collected in a 50-mL volumetric flask to a constant volume for the measurement of soluble sugar and sucrose. Then, starch in the insoluble precipitate was reacted with HCl in boiling water and neutralized with the same amount of NaOH. The solutions of soluble sugar and starch were separately diluted and quantified at 625 and 620 nm with a microplate reader (Epoch 2 Microplate Spectrophotometer, BioTek Corporation, Vermont, USA).

Stem SCs were also measured with three independent biological replicates at the silking stage. One gram of dry stem sample below the ear from the same plant to determine NSCs was weighed from each plot to measure the neutral detergent fiber (NDF), acid detergent fiber (ADF), and acid detergent lignin (ADL) according to the Van Soest procedure (Van Soest et al., 1991). ADL content indicated lignin content; the difference between ADF and ADL was calculated to determine the cellulose content. The hemicellulose content was calculated as the difference between NDF and ADF.

### NSCs in grains

At 12 DAS in 2021, two ears (of average plant height, stem diameter, and ear length within two rows of maize plants) were taken from each plot. A randomly selected row of kernels in each ear were immediately frozen in liquid nitrogen and then mechanically ground to determine the carbohydrate content, enzyme activities, and polyamine content. NSCs containing glucose, fructose, and sucrose in kernels were determined by the HPLC method with modifications (Hanft and Jones, 1986; Shen et al., 2020). One hundred mg of the freeze-dried sample was extracted in 5 mL of distilled water at 70°C for 2 h and centrifuged at 1900 *g* for 15 min. The supernatant containing the soluble sugars was poured off, evaporated to dryness, and re-dissolved with 0.5 mL of distilled water, and 1.5 mL acetonitrile was added. The extracted solution was filtered through a 0.22-μm Millipore filter and then injected into the HPLC system. HPLC analysis was conducted with a Waters 2414 Refractive Index Detector, a Waters 600 Pump, a Waters 600 Controller, and Waters XBridy Amide Columns. The mobile phase was 80% acetonitrile and 20% ultrapure water (containing 0.1% ammonium hydroxide). The flow rate of the pump was set to 1.0 ml min^-1^. NSCs were quantified using external standards of glucose, fructose, and sucrose (Genepioneer Biotechnologies, Nanjing, China). Additionally, the remaining insoluble pellet after extraction was used to determine the starch content, as described above.

### Sucrose- and starch-related enzymatic activities in kernels

Measurements of adenosine diphosphate pyrophosphorylase (AGPase), sucrose synthase (SUS), and cell wall acid invertase (CWIN) activities were determined according to Nakamura et al. (1989). One hundred mg of freeze-dried ground grains in each plot (taken at 12 DAS) were homogenized in a precooled buffer containing 5 mL of 50 mM (HEPES)-NaOH, 10 mM MgCl_2_, 2 mM EDTA, 50 mM 2-mercaptoethanol, 12.5% glycerol, and 5% PVP-40; and 1 M NaCl was also added when extracting CWIN. The homogenate was centrifuged at 15,000 *g* for 15 min at 4°C. The activity of the crude enzyme extracts was determined with the assay kits of AGPase, SUS and CWIN (serial number: YX-E21914P, YX-E22143P and YX-E22311P, Genepioneer Biotechnologies, Nanjing, China).

### Spermine, spermidine, putrescine, and cadaverine in kernels

Polyamines were extracted from 1 g of freeze-dried kernels in each plot (taken at 12 DAS) in 5 mL of 5% perchloric acid at 4°C for 60 min. The samples were then centrifuged for 30 min at 23,000 *g* at 4°C. The standards of spermine (Spm), spermidine (Spd), putrescine (Put), and cadaverine (Cad) were purchased from Genepioneer Biotechnologies Company (Nanjing, China). Benzoylation and determination of standard samples and plant extracts were performed according to Feng et al. (2011). 1 ml 2 M NaOH was mixed with 500 μL HClO^4^ extract. Then added 7 μL benzoyl chloride and vortexed for 20 s. After 37 °C water bath for 20 min, added 2 mL saturated NaCl^2^. Benzoyl-polyamines was extracted in 2 ml diethyl ether and centrifuged at 1500 *g* for 5 min. The 1 mL of ether after evaporation and drying under warm air dissolved in 100 μL of methanol and vortexed for 20 s. Treatment results for the 40 μL standard were similar. HPLC analysis used a Waters programmable liquid chromatograph with a 600 controller and dual wavelength absorbance detector (Waters 2487). Isokinetic elution was performed in 60:40 methanol: water at a flow rate of 0.7 mL min^-1^. The 10 μL of benzoylated extracts were eluted by reversed-phase (C18) column at 30 °C and were detected at 254 nm.

### Photosynthetic rate, transmittance, and stem-breaking force

The net photosynthetic rate (Pn) of ear leaves (three plants per plot) was measured with a photosynthetic instrument (Li-Cor 6400, Li-Cor Bioscience, Nebraska, USA), which was obtained from 10:00 am to 12:00 am under a leaf temperature of 30°C and an irradiation of 1200 μ mol m^−2^ s^−1^ during the silking period in 2021.

Photosynthetically active radiation (PAR) was measured at the top (10 cm above maize canopy), ear position, and bottom (10 cm above ground) between two rows of plants at 25 DAS with three sites in each plot in both years using the LAI CEPTOMETER (LP-80).


$$\text{PAR transmittance }=\frac{\text{intercepted light of the bottom or ear layer}}{\text{incident light at the top}}$$

The stem-breaking force was determined at 30 DAS with three plants in each plot. The dynamometer (ELK-300 N, Zhejiang, China) was slowly positioned perpendicular to the ear internode until the stem was parallel to the ground. The maximum value obtained in this procedure was recorded as the breaking force.

### Grain yield and yield components

Maize ears (5 m row length times one row) were manually harvested in each plot during the mature period. Firstly, ear number and total fresh weight were determined. Then, ten representative ears were selected in each plot by average fresh weight to determine the row number and kernel number per row. After manually threshing, one thousand kernels were counted with two repetitions and dried at 80°C to a constant weight to determine TKW. Grain yield was calculated based on 14% grain water content.

### Statistical analyses

General linear modeling (GLM) using SPSS 25.0 (SPSS, Inc., Chicago, IL, USA) was applied to analyze the influence of plant density, EC treatment, and their interaction on each variable. Duncan’s multiple comparison method was employed to determine differences using Duncan's significant difference test at the 0.05 level. The determination coefficients and P-values in this manuscript were also calculated by linear regression using SPSS 25.0.

## Results

### Grain yield and yield components

Under D1, D2, and D3, KNE was decreased by 2.4–3.8% and 3.9–9.6% under T1 and T2 in 2020 and by 8.9–10.6% and 9.8–16.0% under T1 and T2 in 2021, respectively, compared with the control. The KNM showed almost identical changes with KNE in this study. T1 and T2 reduced grain yield by 2.9–5.9% and 4.3–10.6% in 2020 and by 4.8–11.4% and 6.5–12.8% in 2021, respectively. However, no significant differences in KNE, KNM, or maize yield were observed between EC treatments at the D4 density in 2020 and 2021. Additionally, in this experiment, TKW did not show significant changes among EC treatments under each density. Moreover, increasing density improved KNM and grain yield but decreased KNE and TKW. Compared to other densities, the yield under D4 was significantly increased by 10.7–48.9% and 7.3–41.6% than other densities in 2020 and 2021, respectively (**Tables 1** and **2**). Grain yield and yield components were significantly affected by year type (Y) and plant density (PD). EC had a significant influence on grain yield and KNE but not on TKW. The interactions of Y×PD and Y×EC significantly affected KNE and TKW, while PD×EC and Y×PD×EC had significant effects on grain yield (**Table 3**).

**Table 1:** Effects of plant growth regulator (EC) application and plant density (PD) on maize grain yield and yield components in 2020.

PD	EC	Yield (t ha^-1^)	ENM	TKW (g)	KNE	KNM
D1	CK	9.16 a	4.78 a	322.8 a	542 a	2440 a
	T1	8.62 b	4.67 a	315.9 a	522 b	2348 b
	T2	8.41 b	4.67 a	308.8 a	521 b	2344 b
	**Mean**	**8.73 D**	**4.70 D**	**315.8 A**	**528 A**	**2377 D**
D2	CK	10.95 a	5.89 a	289.3 a	543 a	3256 a
	T1	10.63 ab	6.11 a	287.6 a	530 a	3180 a
	T2	10.48 b	5.78 a	292.5 a	514 a	3083 a
	**Mean**	**10.69 C**	**5.93 C**	**289.8 B**	**529 A**	**3173 C**
D3	CK	12.42 a	7.44 a	297.9 a	479 a	3590 a
	T1	11.72 b	7.44 a	291.4 a	461 ab	3459 ab
	T2	11.10 c	7.44 a	294.5 a	433 b	3245 b
	**Mean**	**11.75 B**	**7.44 B**	**294.6 B**	**458 B**	**3431 B**
D4	CK	13.17 a	8.89 a	290.1 a	434 a	3907 a
	T1	12.94 a	9.00 a	287.7 a	430 a	3871 a
	T2	12.89 a	8.89 a	286.6 a	430 a	3869 a
	**Mean**	**13.00 A**	**8.93 A**	**288.1 B**	**431 C**	**3882 A**

D1, D2, D3, and D4 indicated plant density at 4.5, 6.0, 7.5, and 9.0 plants m^−2^, respectively; CK, T1, and T2 indicated water control, 2 mL L^−1^ EC (ethephon: cycocel = 3:1), 4 mL L^−1^ EC, respectively. ENM, ear number m^-2^; TKW, thousand kernel weight; KNE, kernel number per ear; KNM, kernel number m^-2^. According to Duncan's significant difference test, the same lowercase letters within a column were not significantly different between the EC treatments (unbolded font) under each density at P< 0.05, and the same uppercase letters within a column were not significantly different among plant densities at *P< 0.05*. Bold values mean average.

**Table 2:** Effects of plant growth regulator (EC) application and plant density (PD) on maize grain yield and yield components in 2021.

PD	EC	Yield (t ha^-1^)	ENM	TKW (g)	KNE	KNM
D1	CK	10.11 a	4.67 a	294.2 a	657 a	2955 a
	T1	9.52 ab	4.67 a	305.6 a	596 b	2680 b
	T2	9.34 b	4.67 a	304.1 a	587 b	2642 b
	**Mean**	**9.66 D**	**4.67 D**	**301.3 A**	**613 A**	**2759 D**
D2	CK	12.28 a	5.89 a	282.0 a	625 a	3748 a
	T1	10.89 b	6.11 a	279.4 a	559 b	3355 b
	T2	10.72 b	5.89 a	292.7 a	525 b	3152 b
	**Mean**	**11.30 C**	**5.96 C**	**284.7 B**	**570 B**	**3418 C**
D3	CK	13.25 a	7.44 a	266.2 a	571 a	4284 a
	T1	12.61 ab	7.33 a	278.2 a	520 b	3898 b
	T2	12.39 b	7.33 a	276.0 a	515 b	3861 b
	**Mean**	**12.75 B**	**7.37 B**	**273.5 C**	**535 C**	**4014 B**
D4	CK	13.91 a	8.78 a	259.9 a	511 a	4602 a
	T1	14.68 a	8.78 a	274.4 a	511 a	4598 a
	T2	13.44 a	8.89 a	264.5 a	486 a	4373 a
	**Mean**	**14.01 A**	**8.81 A**	**266.3 C**	**503 C**	**4524 A**

D1, D2, D3, and D4 indicated plant density at 4.5, 6.0, 7.5, and 9.0 plants m^−2^, respectively; CK, T1, and T2 indicated water control, 2 mL L^−1^ EC (ethephon: cycocel = 3:1), 4 mL L^−1^ EC, respectively. ENM, ear number m^-2^; TKW, thousand kernel weight; KNE, kernel number per ear; KNM, kernel number m^-2^. According to Duncan's significant difference test, the same lowercase letters within a column were not significantly different between the EC treatments (unbolded font) under each density at P< 0.05, and the same uppercase letters within a column were not significantly different among plant densities at *P< 0.05*. Bold values mean average.

**Table 3:** Results of ANOVA on the effects of year (Y), plant density (PD), EC treatments (EC) and their interactions on maize yield, ENM, TKW, KNE and KNM in 2020–2021.

ANOVA	Yield (t ha^-1^)	ENM	TKW (g)	KNE	KNM
Y	96.99***	0.45ns	63.48***	313.84***	306.22***
PD	416.66***	696.36***	49.45***	150.84***	688.15***
EC	27.1***	0.34ns	0.48ns	44.17***	37.32***
Y×PD	1.11ns	0.21ns	4.57**	6.25**	11.98***
Y×EC	0.57ns	0.12ns	5.55**	8.87***	6.86**
PD×EC	2.76*	0.46ns	0.84ns	3.12*	2.47*
Y×PD×EC	2,76*	0.1ns	0.84ns	1.61ns	1.43ns

F values and significance levels are given. ENM, ear number m^-2^; TKW, thousand kernel weight; KNE, kernel number per ear; KNM, kernel number m^-2^. ns means not significant at *P< 0.05*. *, ** and *** indicate significance at *P< 0.05,< 0.01* and *P< 0.001*, respectively.

### LAI and maize photosynthetic characteristics

**Figure 2:**
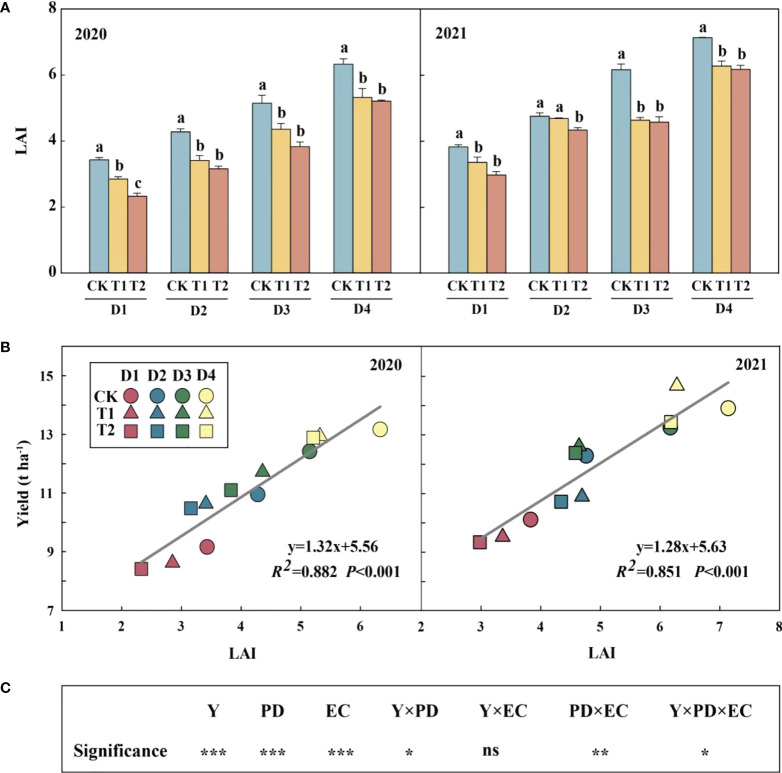
Effects of EC and PD on leaf area index (LAI) in summer maize in both years **(A)**. Different lowercase letters indicate significant differences between EC treatments at each plant density. Correlation of leaf area index (LAI) with yield under EC and PD in both years (*n = 12*) **(B)**. Results of ANOVA on the effects of year **(Y)**, plant density (PD), EC treatments (EC) and their interactions on LAI in 2020–2021 are listed **(C)**. *R^2^* indicates the coefficient of determination. EC, plant growth regulator; PD, plant density; D1, D2, D3, and D4 indicated plant density at 4.5, 6.0, 7.5, and 9.0 plants m^−2^, respectively; CK, T1, and T2 indicated water control, 2 mL L^−1^ EC (ethephon: cycocel = 3:1), 4 mL L^−1^ EC, respectively. ns means non-significant. *R^2^* > 0.4 and *P*< 0.05 indicate a significant relationship. *, ** and *** indicate significant differences at *P< 0.05,< 0.05 and< 0.001* probability levels, respectively. The data used for the regression analysis are the mean values under each treatment.

Year type, PD, EC and their interactions (except for Y×EC) significantly affected LAI (**Figure 2**). Across densities, 15.3–20.3% and 17.6–32.2% in 2020 and 12.1–24.8% and 8.9–25.8% reductions in LAI were observed in 2021 under T1 and T2, respectively, compared to CK. Meanwhile, increasing density significantly increased LAI, which had a significant positive correlation with yield in both years (**Figure 2B**).

The net photosynthetic rate of the ear leaf was increased with a high EC dose but had a decreasing tendency with the increase of plant density (**Figure S1**). Specifically, the net photosynthetic rate was improved by 9.6% and 19.1% under T1 and T2, respectively, in comparison to CK. Similarly, both ear and bottom transmittances increased under EC treatment in both years (**Figure S2**). The ear transmittance of T2 increased significantly by 14.9% in 2020 and by 18.7% in 2021, compared with the control.

### Silk number per ear

**Figure 3:**
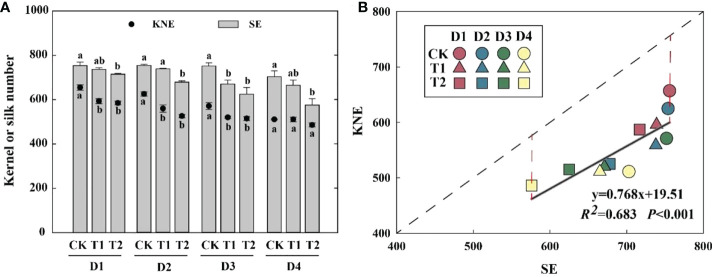
Effects of EC and PD on KNE and SE in summer maize in 2021 **(A)**. Different lowercase letters indicate significant differences between EC treatments at each plant density. Correlation between SE and KNE under EC and PD in 2021 (*n = 12*) **(B)**. Solid lines indicate linear regression, and dashed lines indicate 1:1. *R^2^* indicates the coefficient of determination. *R^2^* > 0.4 and *P*< 0.05 indicate a significant relationship. EC, plant growth regulator; PD, plant density; D1, D2, D3, and D4 indicated plant density at 4.5, 6.0, 7.5, and 9.0 plants m^−2^, respectively; CK, T1, and T2 indicated water control, 2 mL L^−1^ EC (ethephon: cycocel = 3:1), 4 mL L^−1^ EC, respectively. KNE, kernel number per ear; SE, silk number per ear. The data used for the regression analysis are the mean values under each treatment.

EC reduced KNE and SE in 2021 (**Figure 3A**). Compared with the control, the SE of T1 and T2 was decreased by 2.0–10.7% and 5.2–18.1%, respectively, which was significantly positively correlated with KNE (**Figure 3B**). The differences between the theoretical and actual KNE under each treatment reached 114–156 kernels, indicating that total florets were enough for kernel setting.

### Nonstructural carbohydrates

**Figure 4:**
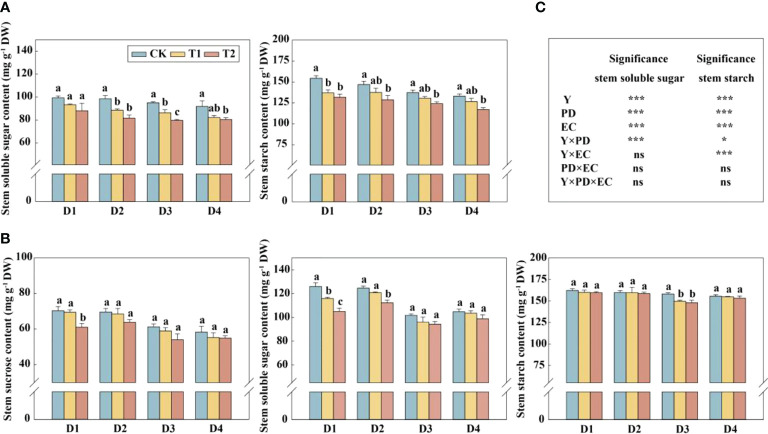
Effects of EC and PD on soluble sugar and starch in the stems of summer maize in 2020 **(A)**. Effects of EC and PD on sucrose, soluble sugar, and starch in the stems of summer maize in 2021 **(B)**. Results of ANOVA on the effects of year **(Y)**, plant density (PD), EC treatments (EC) and their interactions on soluble sugar and starch in the stems in 2020–2021 are listed **(C)**. Different lowercase letters indicate significant differences between EC treatments at each plant density. ns means non-significant. * and *** indicate significant differences at *P< 0.05 and< 0.001* probability levels, respectively. EC, plant growth regulator; PD, plant density; D1, D2, D3, and D4 indicated plant density at 4.5, 6.0, 7.5, and 9.0 plants m^−2^, respectively; CK, T1, and T2 indicated water control, 2 mL L^−1^ EC (ethephon: cycocel = 3:1), 4 mL L^−1^ EC, respectively.

**Figure 5:**
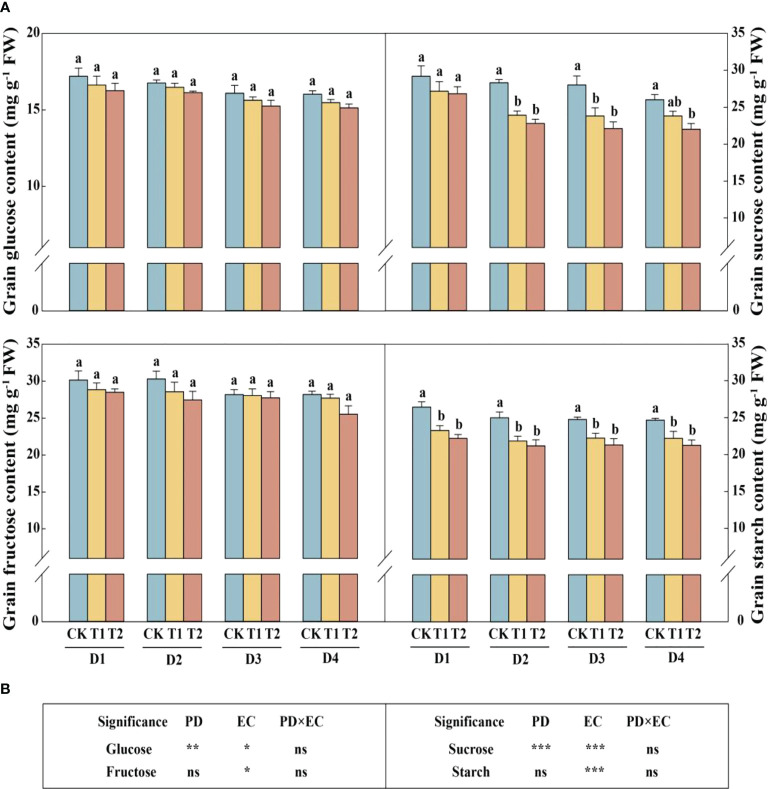
Effects of EC and PD on glucose, sucrose, fructose, and starch in the grains of summer maize in 2021 **(A)**. Results of ANOVA on the effects of plant density (PD), EC treatments (EC) and their interaction (PD×EC) on glucose, sucrose, fructose, and starch in the grains in 2021 are listed **(B)**. Different lowercase letters indicate significant differences between EC treatments at each plant density. ns means non-significant. *, ** and *** indicate significant differences at *P< 0.05, < 0.01*, and *< 0.001* probability levels, respectively. EC, plant growth regulator; PD, plant density; D1, D2, D3, and D4 indicated plant density at 4.5, 6.0, 7.5, and 9.0 plants m^−2^, respectively; CK, T1, and T2 indicated water control, 2 mL L^−1^ EC (ethephon: cycocel = 3:1), 4 mL L^−1^ EC, respectively.

In this study, year type, PD, EC significantly affected soluble sugar and starch in the stem (**Figure 4**). Across the densities, the soluble sugar and starch concentrations of stems were significantly reduced by 12.4%–17.1% (except 4.5 plants m^−2^) and 9.6%–12.4% in 2020, respectively, under T2 treatment (**Figure 4A**). The concentrations of sucrose and soluble sugar in the stem (across all the plant densities) were prominently decreased by 9.9% and 10.2%, respectively, under T2 treatment in 2021 (**Figure 4B**). The glucose, sucrose, fructose, and starch concentrations in grains with increasing EC concentrations under the same density treatment showed a downward trend in 2021 (**Figure 5A**). Furthermore, EC significantly affected NSCs in grains (**Figure 5B**). Across densities, kernel sucrose concentrations under T1 and T2 were significantly decreased by 11.5% and 15.9%, respectively, and the starch concentrations under T1 and T2 were reduced by 11.1% and 14.7%, respectively. The glucose and fructose concentrations of the kernels were significantly lower at T2, but not at T1, compared to CK.

### Polyamine and sink activity

**Figure 6:**
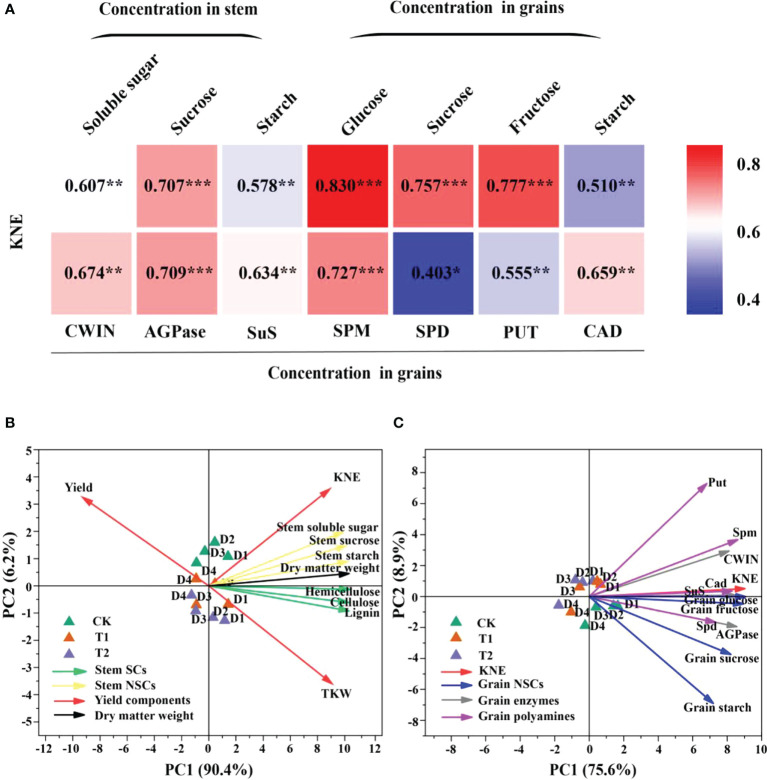
Relationships between soluble sugar, sucrose, and starch in stem and glucose, sucrose, fructose, starch, cell wall acid invertase (CWIN), adenosine diphosphate pyrophosphorylase (AGPase), sucrose synthase decomposition direction (SuS), spermidine (Spm), spermidine (Spd), putrescine (Put), and cadaverine (Cad) in grains with KNE in 2021 (*n = 12*) **(A)**. The numbers in the figure represent *R^2^*, the coefficient of determination. *R^2^* > 0.4 and *P*< 0.05 indicate a significant relationship. *, **, and *** indicate significant differences at *P< 0*.*05*, *0*.*01*, and *0*.*001*, respectively. KNE, kernel number per ear. Principal component analysis (PCA) of yield components, stem NSCs, stem SCs and stem dry matter weight **(B)**, and KNE, grain NSCs, grain enzymes and grain polyamines **(C)** in 2021. Vectors represent trait factor loading coordinates for PC1 and PC2. D1, D2, D3, and D4 indicated plant density at 4.5, 6.0, 7.5, and 9.0 plants m^−2^, respectively; CK, T1, and T2 indicated water control, 2 mL L^−1^ EC (ethephon: cycocel = 3:1), 4 mL L^−1^ EC, respectively. The data used for the regression analysis are the mean values under each treatment.

Carbon metabolism-related enzymes, CWIN and AGPase, were significantly affected by EC, while PD significantly changed the activities of CWIN and SuS (**Table 4**). CWIN, AGPase, and SuS activities were reduced by 4.1%, 4.2%, and 3.9%, respectively, under T1 and by 6.2%, 6.2%, and 5.8%, respectively, under T2, compared to CK (average value; **Table 4**). In addition, PD and EC significantly affected polyamines, except for the effect of EC on Put (**Table 5**). Spm, Spd and Cad concentrations were slightly decreased by 7.6%, 2.9% and 11.1% under T2 (average value; **Table 5**). Furthermore, the polyamines and enzyme activities showed significant positive correlations with KNE (**Figure 6A**). Additionally, significant positive correlations were observed between NSC and KNE in both stems and grains (**Figure 6A**).

**Table 4:** Effects of EC and PD on cell wall acid invertase (CWIN), adenosine diphosphate pyrophosphorylase (AGPase), sucrose synthase decomposition direction (SuS) in grains of summer maize in 2021.

PD	EC	CWIN(μ g reducing sugar g^-1^ FW min^-1^)	AGPase(n mol NADPH g^-1^ FW min^-1^)	SuS(μ g sucrose g^-1^ FW min^-1^)
D1	CK	1468.37±47.47 a	558.65±3.40 a	548.28±19.13 a
	T1	1431.28±62.01 a	521.93±17.50 a	549.17±19.32 a
	T2	1396.94±66.20 a	511.88±14.36 a	538.88±16.99 a
D2	CK	1446.68±32.39 a	556.90±14.41 a	546.63±21.21 a
	T1	1366.06±48.57 a	522.20±9.57 a	545.53±22.56 a
	T2	1310.47±41.53 a	509.25±22.42 a	524.55±6.20 a
D3	CK	1422.20±43.74 a	525.44±21.17 a	538.65±21.22 a
	T1	1399.85±39.42 a	522.62±7.57 a	498.62±1.69 a
	T2	1380.89±57.27 a	515.63±21.25 a	494.82±13.95 a
D4	CK	1320.40±42.77 a	520.75±24.49 a	536.08±16.58 a
	T1	1226.84±45.05 a	503.87±13.77 a	492.57±16.62 a
	T2	1215.89±16.62 a	490.74±16.19 a	484.58±22.46 a
ANOVA				
PD		***	ns	*
EC		*	*	ns
PD×EC		ns	ns	ns

D1, D2, D3, and D4 indicated plant density at 4.5, 6.0, 7.5, and 9.0 plants m^−2^, respectively; CK, T1, and T2 indicated water control, 2 mL L^−1^ EC (ethephon: cycocel = 3:1), 4 mL L^−1^ EC, respectively. Different lowercase letters indicate significant differences between EC treatments at each plant density. ns means non-significant. * and *** indicate significant differences at *P< 0.05* and *< 0.001* probability levels, respectively. EC, plant growth regulator; PD, plant density.

**Table 5:** Effects of EC and PD on spermidine (Spm), spermidine (Spd), putrescine (Put), and cadaverine (Cad) in grains of summer maize in 2021.

PD	EC	Spm (nmol g^-1^ FW)	Spd (nmol g^-1^ FW)	Put (nmol g^-1^ FW)	Cad (nmol g^-1^ FW)
D1	CK	141.25±2.51 a	112.70±0.78 a	3549.04±81.05 a	1.67±0.08 a
	T1	134.32±3.20 ab	109.56±1.57 a	3548.46±30.62 a	1.60±0.04 a
	T2	127.24±1.28 b	108.52±3.07 a	3545.52±6.57 a	1.54±0.13 a
D2	CK	139.99±3.47 a	113.58±1.16 a	3466.77±3.75 a	1.75±0.02 a
	T1	139.63±5.13 a	112.94±1.80 a	3466.70±29.56 a	1.74±0.12 a
	T2	129.73±4.33 a	112.01±0.36 a	3449.26±13.84 a	1.42±0.07 b
D3	CK	122.88±4.29 a	112.03±1.14 a	3468.33±13.44 a	1.49±0.07 a
	T1	118.54±3.90 a	107.09±1.56 a	3457.81±14.79 a	1.39±0.12 a
	T2	118.50±4.54 a	107.89±1.47 a	3455.94±17.84 a	1.40±0.03 a
D4	CK	115.32±5.34 a	110.43±2.96 a	3256.82±29.42 a	1.47±0.13 a
	T1	112.81±5.10 a	108.10±1.00 a	3235.79±48.80 a	1.47±0.07 a
	T2	104.74±2.67 a	107.30±1.20 a	3235.72±97.52 a	1.31±0.08 a
ANOVA					
PD		***	*	***	**
EC		**	*	ns	*
PD×EC		ns	ns	ns	ns

D1, D2, D3, and D4 indicated plant density at 4.5, 6.0, 7.5, and 9.0 plants m^−2^, respectively; CK, T1, and T2 indicated water control, 2 mL L^−1^ EC (ethephon: cycocel = 3:1), 4 mL L^−1^ EC, respectively. Different lowercase letters indicate significant differences between EC treatments at each plant density. ns means non-significant. *, ** and *** indicate significant differences at *P< 0.05, < 0.01* and *< 0.001* probability levels, respectively. EC, plant growth regulator; PD, plant density.

### Stem lodging resistance index and structural carbohydrates

**Figure 7:**
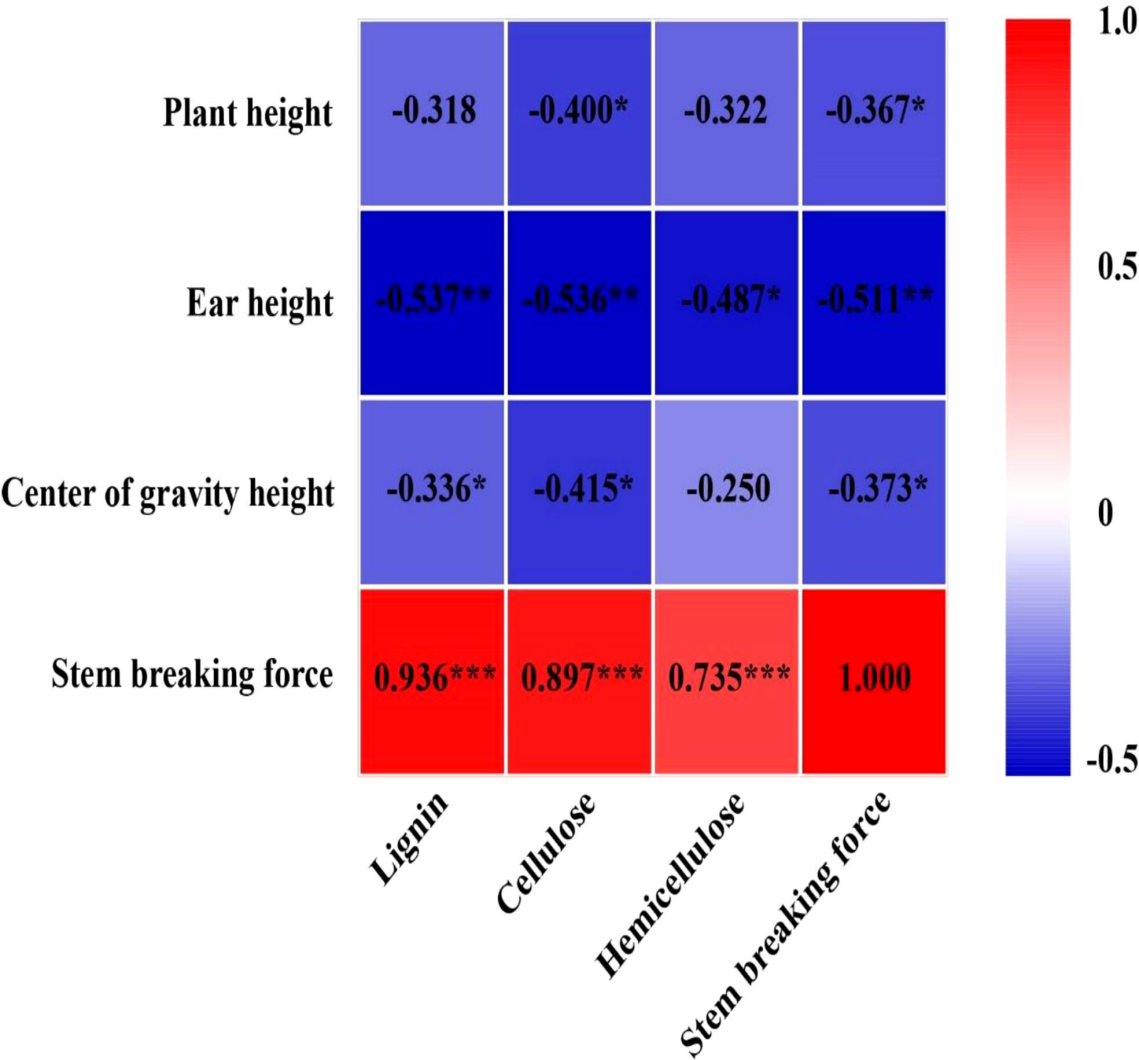
Relationship between cellulose, hemicellulose, lignin, and stem-breaking force with plant height, ear height, center of gravity height, and stem-breaking force in 2021 (*n = 12*). The numbers in the figure represent *R^2^*, the coefficient of determination. “−” represents a negative correlation between the two indicators. *R^2^* > 0.4 and *P*< 0.05 indicate a significant relationship. *, ** and *** indicate significant differences at *P< 0*.*05, 0.01* and *0*.*001*, respectively. The data used for the regression analysis are the mean values under each treatment.

**Figure 8:**
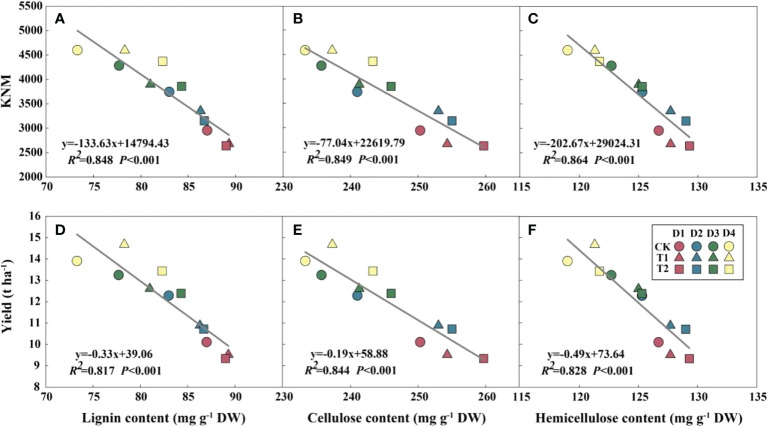
Relationships between structural carbohydrates with kernel number m^-2^ (KNM) **(A–C)** and grain yield **(D–F)** in 2021 (*n = 12*). *R^2^* indicates the coefficient of determination. *R^2^* > 0.4 and *P*< 0.05 indicate a significant relationship. EC, plant growth regulator; PD, plant density; D1, D2, D3, and D4 indicated plant density at 4.5, 6.0, 7.5, and 9.0 plants m^−2^, respectively; CK, T1, and T2 indicated water control, 2 mL L^−1^ EC (ethephon: cycocel = 3:1), 4 mL L^−1^ EC, respectively. The data used for the regression analysis are the mean values under each treatment.

EC significantly reduced plant height, ear height, and center of gravity height in both years, particularly in the T2 treatment (**Figure S3**). Conversely, the diameter of the basal internode increased under EC treatments (**Figure S4**). Compared with CK, EC slightly improved the lignin, cellulose, and hemicellulose concentrations in the stem (**Table 6**). Correspondingly, the stem-breaking force was enhanced under EC compared to that of the control, notably under T2 treatment (**Figure S5**). Additionally, the stem-breaking force was significantly positively correlated with cellulose, hemicellulose, and lignin, while SC concentrations and stem-breaking force showed significant negative correlations with plant, ear, and center of gravity height (**Figure 7**). Moreover, both KNM and yield were significantly negatively correlated with the SC of the stem, suggesting that increased SC with enhanced lodging resistance had an adverse effect on kernel setting when lodging was absent and EC was applied (**Figure 8**).

**Table 6:** Effects of EC and PD on lignin, cellulose, and hemicellulose in the basal stem below the ear in 2021.

PD	EC	Lignin (mg g^-1^ DW)	Cellulose (mg g^-1^ DW)	Hemicellulose (mg g^-1^ DW)
D1	CK	87.00±1.53 a	250.33±13.38 a	126.67±2.60 a
	T1	89.33±3.18 a	254.33±10.84 a	127.67±2.91 a
	T2	89.00±1.15 a	259.67±11.26 a	129.33±4.26 a
D2	CK	83.00±1.53 a	241.00±12.00 a	125.33±2.73 a
	T1	86.33±2.03 a	253.00±11.79 a	127.67±2.85 a
	T2	86.67±2.03 a	255.00±9.87 a	129.00±4.62 a
D3	CK	77.67±1.76 a	235.67±6.17 a	122.67±6.17 a
	T1	81.00±1.73 a	241.33±10.27 a	125.00±1.73 a
	T2	84.33±2.19 a	246.00±4.04 a	125.33±2.03 a
D4	CK	73.33±1.67 b	233.33±4.81 a	119.00±3.79 a
	T1	78.33±1.20 a	237.33±9.24 a	121.33±3.84 a
	T2	82.33±1.33 a	243.33±8.25 a	121.67±6.33 a
ANOVA				
PD		***	ns	ns
EC		**	ns	ns
PD×EC		ns	ns	ns

D1, D2, D3, and D4 indicated plant density at 4.5, 6.0, 7.5, and 9.0 plants m^−2^, respectively; CK, T1, and T2 indicated water control, 2 mL L^−1^ EC (ethephon: cycocel = 3:1), 4 mL L^−1^ EC, respectively. Different lowercase letters indicate significant differences between EC treatments at each plant density. ns means non-significant. ** and *** indicate significant differences at *P< 0.01* and *< 0.001* probability levels, respectively. EC, plant growth regulator; PD, plant density.

### Principal component analysis

Principal component analysis (PCA) analyses for maize grown under different plant densities and EC treatments were performed visualizing the associations between the yield components, stem SCs, stem NSCs and dry matter weight (**Figure 6B**), and between KNE, grain NSCs, grain enzymes and grain polyamines in 2021 (**Figure 6C**). Principal component 1 (PC1) and principal component 2 (PC2) described 90.4 and 6.2% variability among the variables in **Figure 6B**, respectively. For **Figure 6C**, 75.6 and 8.9% of variability was described by PC1 and PC2, respectively. A smaller acute angle (< 90°) between loading vectors means a stronger correlation between variables. Stem NSCs were clustered closer to SCs, and SCs were in opposite direction of yield. Spd, Spm and Cad were located close to grain glucose, fructose, and sucrose, also positively correlated with CWIN, AGPase, and SuS activities (**Figure 6**).

## Discussion

To our knowledge, previous studies which enhanced lodging resistance with PGRs usually focused on lodging indexes and final yield. The physiological mechanism involved in kernels setting and yield received less attention (Zhang et al., 2014; Ahmad et al., 2018; Ahmad et al., 2020; Huang et al., 2021; Zhao et al., 2021; Ren et al., 2022). In this study, trade-off between NSC/SC and grain yield was assessed. The hypothesis that NSC and SC in stems were responsible for kernel number and yield was demonstrated. High SC enhanced lodging resistance but had adverse effects on kernel number and grain yield. The underlying physiological mechanism that EC affected kernel setting was also elucidated. EC reduced source supply thus decreasing sink activity and polyamines, finally showed a negative effect on kernel number and grain yield without lodging.

### Decreased LAI restricted carbohydrate supply despite plant morphology was optimized

Optimized canopy structure with increased plant density could improve light interception and enhance photosynthetic capacity (Maddonni et al., 2001). A suitable LAI was pivotal to guarantee higher yield in maize (Liu et al., 2017; Bonelli and Andrade, 2019). In this study, we also demonstrated that maize with enough LAI showed yield advantage (**Figure 2**). Leaf area beyond or below the critical LAI was inexorably detrimental for crop growth (Bonelli and Andrade, 2019). **Figure 2** showed a linear relationship between yield and LAI, probably because of relative small sample size or the LAI had not reached the maximum value. EC, as well as numerous PGRs, optimizes plant canopy morphology and structure (Xu et al., 2004; Zhao et al., 2021). Spraying EC around the stem elongation stage could obtain a triangular plant type for a more uniform light distribution within the canopy (Huang et al., 2017). Correspondingly, photosynthesis and transmittance at the bottom and ear in maize were ameliorated (**Figures S1**, **S2**). This facilitated carbohydrate fixation in the leaves and carbohydrate storage in the stem for grain growth (Liu et al., 2019; Yan et al., 2021). However, the decrease in NSCs in the stem indicated that even though the canopy structure was optimized with EC, it still could not compensate for the LAI decline, thus ultimately affecting the formations of the kernel number and yield (**Figure 2**). Shekoofa and Emam (2008) reported application of PGR was associated with reductions in leaf area development, crop growth rate, and photoassimilate. Ren et al. (2022) indicated that LAI played a critical role in determining kernel number and yield, in which spraying PGR reduced LAI and grain yield. However, PGR could increase kernel number and yield even though leaf area was reduced with lodging occurring (Zhang et al., 2014). Under high density (9.0 plants m^−2^), relatively high LAI and yield were obtained by spraying EC, even though TKW and KNE were the lowest, in agreement with the results of previous research (Ren et al., 2022). Consequently, spraying PGR to prevent lodging would reduce LAI and grain yield, but high density could maintain trade-off between lodging and yield.

### EC inhibited silk growth and reduced NSC supply thus inhibiting kernel establishment

Generally, female floret initiations start from 8^th^- to 13^th^- leaf stage (Gonzalez et al., 2019), EC applications at the early growth stage (V8) possibly inhibit or damage floret initiation, thus decreasing KN per ear (Zhao et al., 2022). In this study, the decrease in SE also revealed that floret differentiation was inhibited by the EC treatment (**Figure 3**). However, previous research reported that spraying a PGR at V7 (the same stage as in our study) increased the kernel number (Zhang et al., 2014; Xue et al., 2016), suggesting that the reduced kernel number was independent of the number of the initial ovary. Many studies observed that kernel number is determined by assimilate production during the critical period bracketing silking (Andrade et al., 1999; Gao et al., 2018; Gao et al., 2020) and the proportion of assimilate that is partitioned to the ear in maize (Andrade et al., 1999; Borrás and Vitantonio-Mazzini, 2018). Moreover, the final florets (emerged silks) were obviously higher than the final kernels in this study (**Figure 3**). It seems that the kernel number is more related to plant growth and biomass partitioning around the fertilization stage (Oury et al., 2016b). Hence, the emerged silks were significantly reduced by EC, but the kernel number might not be determined by floret differentiation.

Assimilates partitioned to ear/ grain required related metabolic enzymes to be unloaded and synthesized starch, which contributed to kernel growth and develop (Shen et al., 2020). Our results showed that CWIN, SuS, and AGPase were positively correlated KNE (**Figure 6**). Research has shown that weak CWIN activity results in reduced or obstructed sucrose delivery (Shen et al., 2018; Ruan, 2022). In addition, transcript downregulation of sucrose synthase genes, consistent with enzymatic activities, results in grain abortion (McLaughlin and Boyer, 2004). The expression of AGPase can stimulate the reproductive organs in the early development stage and promote the accumulation of starch in ovarian tissue, thereby promoting the abortion of established kernels when AGPase activity was low (Tuncel and Okita, 2013). Increase in sucrose with injection could improve relative transcript abundance of CWIN and soluble invertase and activity of these enzymes (Zinselmeier et al., 1999; McLaughlin and Boyer, 2004). These indicated that reduced source supply might reduce the enzymes activities mentioned above. The results of PCA also showed that these enzymes activities were close to soluble sugar (**Figure 6C**). However, previous studies were usually conducted under stressful conditions (McLaughlin and Boyer, 2004; Slewinski, 2012; Shen et al., 2020), and other signal (e.g., drought signal ABA) also down-regulated the enzymes activity. In this study, the main change under EC was the reduction of NSC supply owing to lower LAI, which inhibited sink activity and finally affected the kernel number. Moreover, EC was applied at V7 (around 30 d before tasseling), and the impact of EC generally lasted for 15 days (Gao et al., 2017), i.e., exogenous or/ and endogenous hormones would not trigger sugar-metabolizing enzymes changes. Therefore, the variation in grain sink activity during the lag period was not directly affected by EC, which was regulated by the reduced assimilate supply (LAI) under PGRs (Gao et al., 2017). As discussed above, optimized canopy and increased photosynthesis do not compensate for source loss caused by LAI decline, which plays a dominant role in kernel set and grain yield.

Additionally, our results indicated that polyamines were positively correlated with KNE (**Figure 6A**). Previous research also reported that low polyamine content could cause decrease in DNA content and mitotic rate in the endosperm, thereby affecting grain growth and development (Liang and Lur, 2002). Meanwhile, aborted kernels were found to have a significant lower polyamine content than normal kernels after pollination (Liang and Lur, 2002). Moreover, abundant glucose in kernels can accelerate polyamine biosynthesis (Feng et al., 2011). PCA analysis in this study also indicated that glucose and fructose were positively correlated with Cad and Spd (**Figure 6C**). EC reduced the source supply, which accompanied with lower CWIN activity thus decreasing glucose and fructose in the kernels. Correspondingly, the polyamines in the kernels decreased in comparison to CK (**Table 5**). Taken together, we concluded that EC reduced NSC supply owing to decreasing LAI, thus reducing sink activity and polyamines, which showed adverse effects on kernel number and grain yield. Additionally, in this density experiment, KNE did not show positive relationship with grain yield across all the densities owing to ear number per hectare determining the grain yield. However, a significant positive correlation between KNE and grain yield was detected under each density (**Figure S6**). EC mainly reduced KNE and increase of density could improve KNM and grain yield.

### Stem SCs competed with kernels for non-structural carbohydrates under EC

Proceeding studies have detected that the trade-off was existed between KN formation and lodging resistance in wheat (Piñera-Chavez et al., 2016). In maize, lodging-susceptible cultivars yielded more than lodging-resistant cultivars (Zhang et al., 2022). Moreover, negative correlations between yield and stalk lodging resistance were further confirmed, which suggested developing kernels may compete for assimilates with maize stem elongation (Zhang et al., 2022). Berry et al. (2007) showed that stronger basal stem required more assimilates for support structures. The above research demonstrated the trade-offs between dry matter accumulation and partitioning (or yield) and lodging-associated physical traits. However, specific assimilates (e.g., soluble sugar, SC) changes were unclear. In this study, the application of EC at the V7 stage enhanced lodging resistance, accompanied by an increase of SC, especially under high doses of EC. Both NSC and SC contributed to enhancing lodging resistance (Shah et al., 2021). However, our results indicated EC reduced NSC of stem, suggesting SC played more important role in maize lodging. Zuber et al. (1980) also reported that 50–80% of the strength of maize stem came from its rind. Additionally, the PCA analysis indicated that NSC was positively correlated with SC (**Figure 6B**). SCs (cellulose consisting entirely of glucose monomers and hemicellulose composed of pentose and glucose) were important sinks for photosynthate (Sekhon et al., 2016), i.e, parts of NSCs were substrate for SCs. EC reduced photosynthates but improved SCs, thus NSCs in the stem were reduced. Additionally, across all of the densities, SCs were positively correlated with KNE but negatively correlated with KNM. The main reason was that EC treatments did not significantly increase SCs under each plant density, but significantly reduce grain yield. Meanwhile, density significantly increased grain yield but not reduced cellulose and hemicellulose. NSCs in the stem determined the kernel number, especially when leaf source was inhibited (Slewinski, 2012; Gao et al., 2020). Therefore, we concluded that kernels and stem (mainly SCs) competed for NSC, furtherly revealing the negative relationships between grain yield and lodging resistance when lodging was absent. In regard to morphological traits, lodging-resistance maize presented low plant height, ear height, and center of gravity, accompanied by low biomass and yield (Shah et al., 2021). EC, especially with high dose, also reduced plant height (Figure S3). PGR usually inhibits stem elongation and reduced biomass (Shekoofa and Emam, 2008). However, PGR increased the dry matter allocation to stem (Figure S7), in agreement with those reported in Zhang et al. (2022). Therefore, we suggested enhanced lodging with increased dry matter allocation to stem (total biomass was reduced) and increased SC in the stem, would compete with kernels on carbohydrates, resulting in a negative correlation with kernel number and yield when lodging did not occur.

## Conclusion

Application of EC at V7 decreased LAI and NSCs in the stem, ultimately inhibited kernel set, despite optimizing plant morphology in maize. The activities of carbon metabolism-related enzymes and polyamine concentrations in kernels decreased and showed significant positive relationships with kernel number. Additionally, increasing SCs in stem enhanced lodging resistance with EC, which consumed photosynthates and further reduced NSCs availability in the stem and presented a negative correlation with kernel number and yield. In contrast, increasing plant density under EC treatment could ensure relatively high LAI, enhance lodging resistance, and stabilize high maize yield.

## Data availability statement

The original contributions presented in the study are included in the article/**Supplementary Material**. Further inquiries can be directed to the corresponding authors.

## Author contributions

QT: Conceptualization, Data curation, Resources, Investigation, Software, Visualization, Formal analysis, Writing–original draft. JR, Writing–original draft, Writing–review and editing, Supervision. XD, Supervision. SN, SL, DW, and YZ: Investigation. DB: Formal analysis. YC: Supervision. ZG: Conceptualization, Software, Formal analysis, Writing–original draft, Writing–review and editing, Supervision. All authors contributed to the article and approved the submitted version.

## Funding

This work was supported by National Science Fund of China (32101829), Natural Science Research Project of Higher Education in Hebei Province (BJK2022009), Key Research and Development Program of Hebei Province (20326414D, 21327001D), the Hebei Agriculture Research System (HBCT2018020202), State Key Laboratory of North China Crop Improvement and Regulation (NCCIR2022ZZ-12, NCCIR2021ZZ-09), the Startup Fund of Hebei Agricultural University (YJ201827).

## Conflict of interest

The authors declare that the research was conducted in the absence of any commercial or financial relationships that could be construed as a potential conflict of interest.

## Publisher’s note

All claims expressed in this article are solely those of the authors and do not necessarily represent those of their affiliated organizations, or those of the publisher, the editors and the reviewers. Any product that may be evaluated in this article, or claim that may be made by its manufacturer, is not guaranteed or endorsed by the publisher.
