# Supplementary material for: Reduced stem nonstructural carbohydrates caused by plant growth retardant had adverse effects on maize yield under low density
Source: Front Plant Sci. 2022 Oct 20;13:1035254. doi: 10.3389/fpls.2022.1035254 (PMC9632278; doi:10.3389/fpls.2022.1035254)
Supplement: Supplementary file 1 [file Table_1.docx]

***Supplementary Material***

**Supplementary Figure 1**


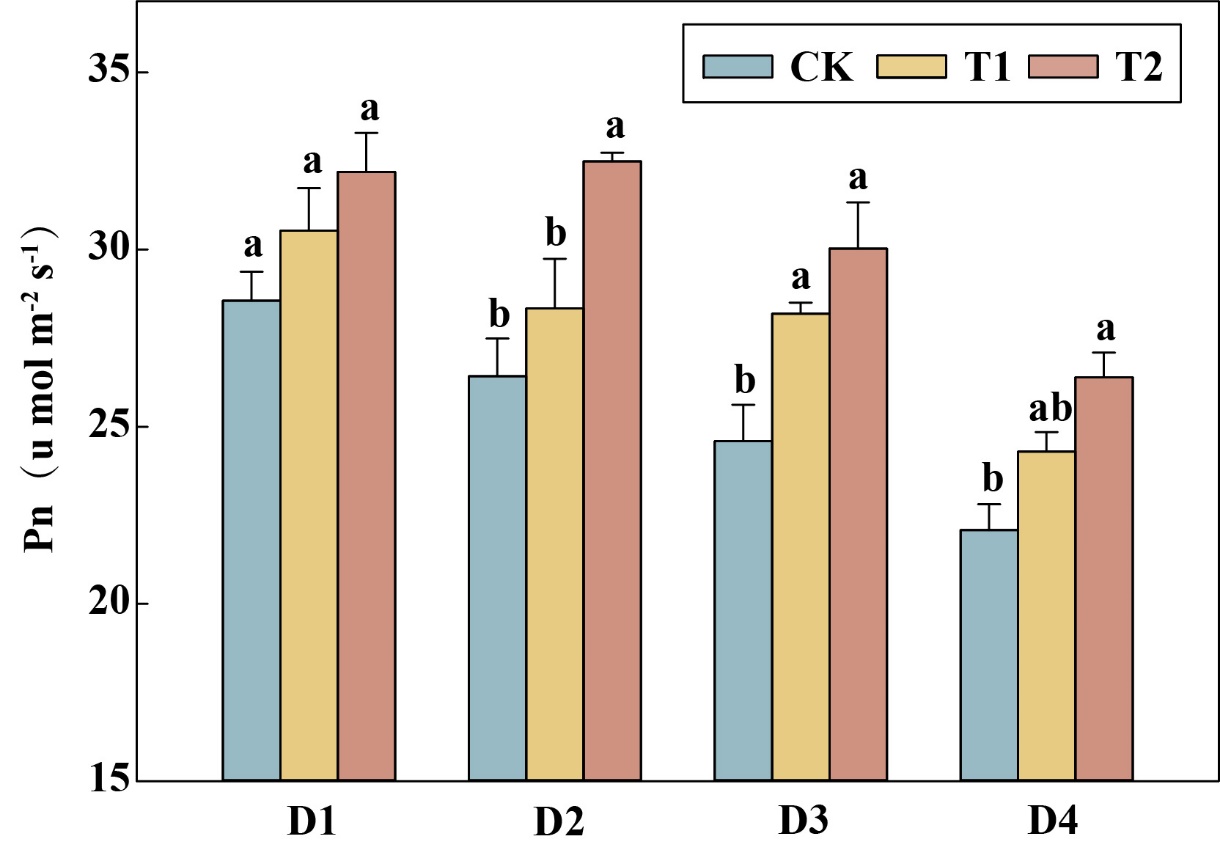


Effect of EC and PD on the net photosynthetic rate of ear position during the silking period in 2021. Different lowercase letters indicate significant differences between EC treatments at each plant density. EC, plant growth regulator; PD, plant density.

**Supplementary Figure 2**
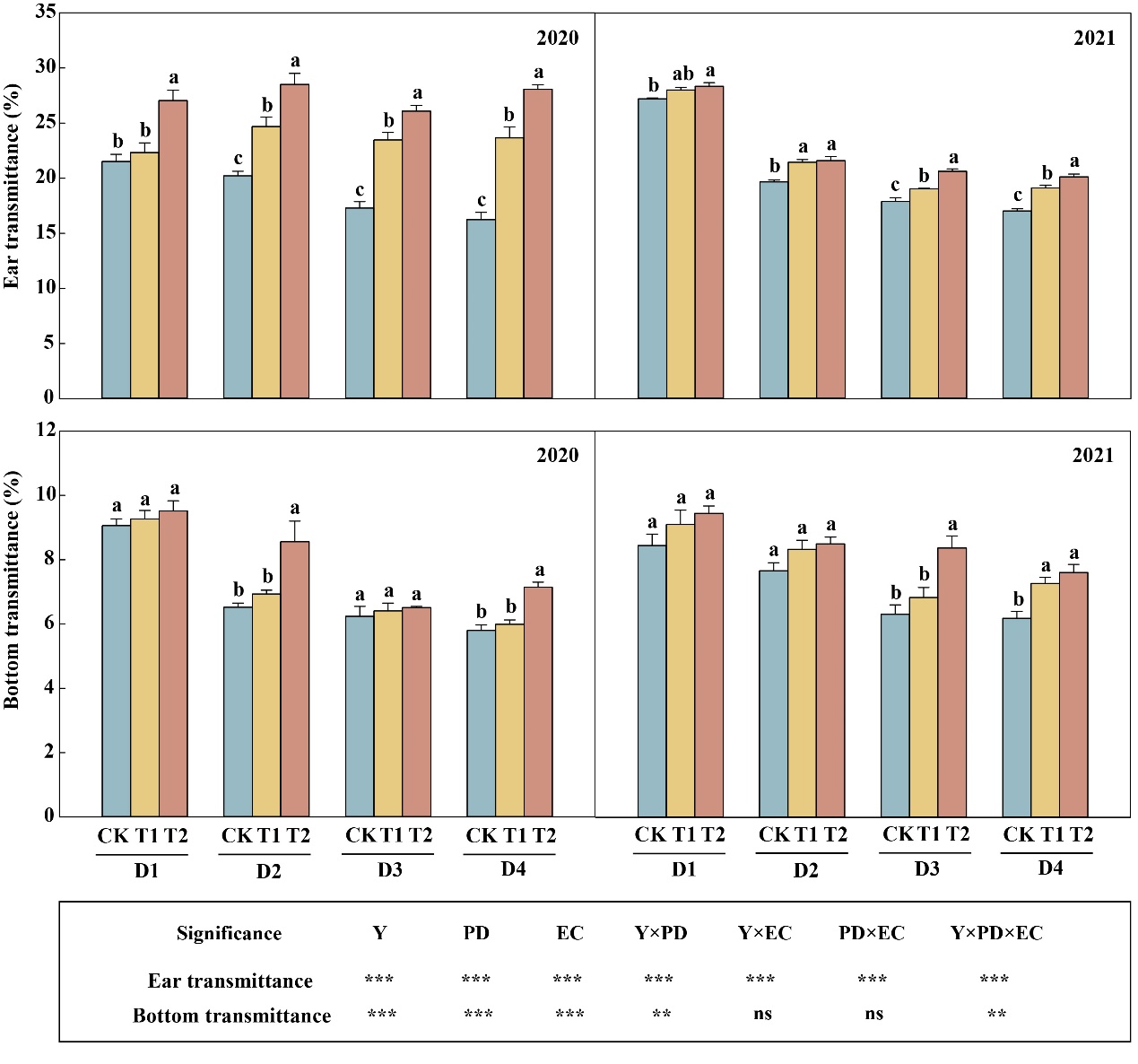


Effect of EC and PD on transmittance at the bottom and at the ear at 25 DAS in both years. Different lowercase letters indicate significant differences between EC treatments at each plant density. EC, plant growth regulator; PD, plant density.

**Supplementary Figure 3**
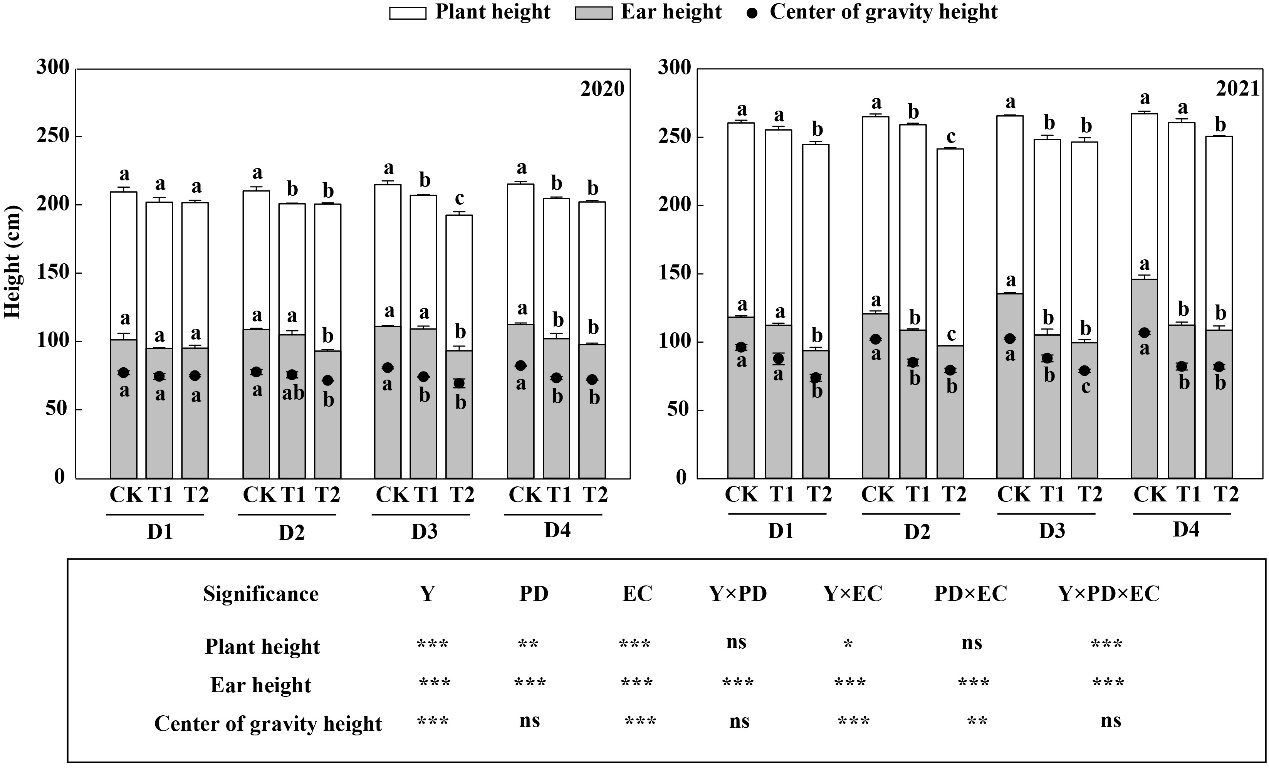


Effect of DC and PD on the morphological structure of summer maize plants in this study. And results of ANOVA on the effects of year (Y), plant density (PD), EC treatments (EC) and their interactions (PD×EC) on the morphological structure of summer maize plants in 2020–2021 are listed. Different lowercase letters indicate significant differences between EC treatments at each plant density. ns means non-significant. *, ** and *** indicate significant differences at *P < 0.05, < 0.01 and < 0.001* probability levels, respectively. EC, plant growth regulator; PD, plant density.

**Supplementary Figure 4
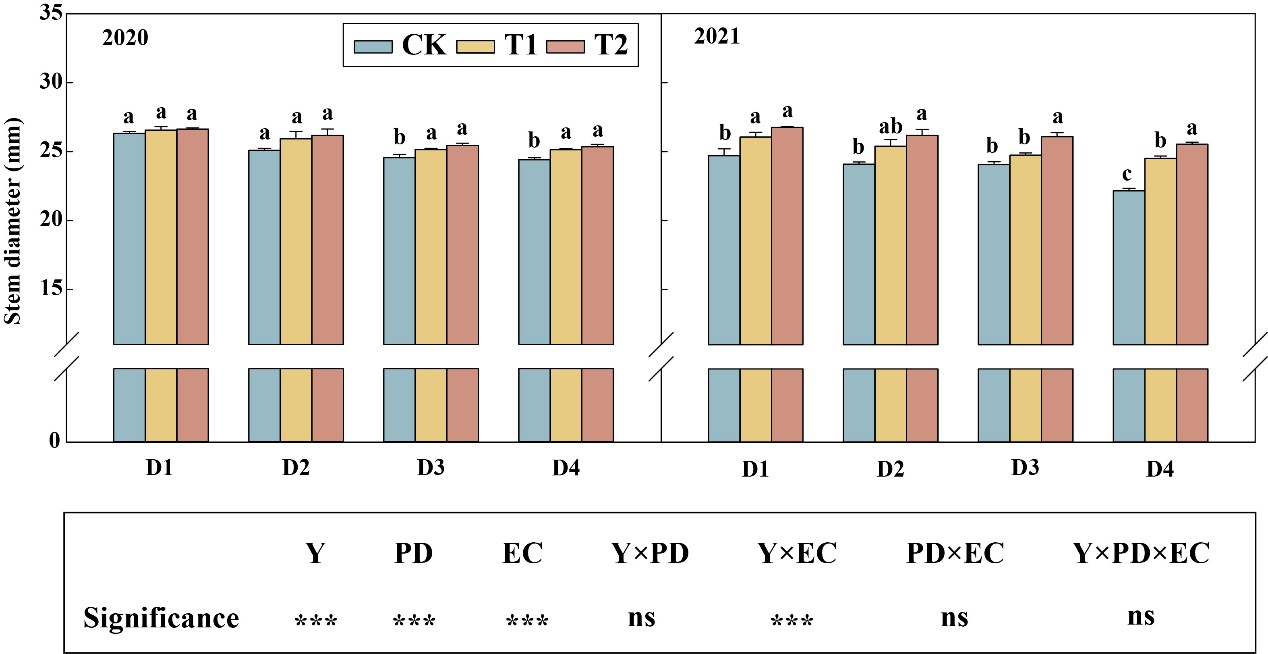
**

Effect of EC and PD on the basal first internode stem diameter in both years. And results of ANOVA on the effects of year (Y), plant density (PD), EC treatments (EC) and their interactions (PD×EC) on the basal first internode stem diameter in 2020–2021 are listed. Different lowercase letters indicate significant differences between EC treatments at each plant density. ns means non-significant. *** indicates significant differences at *P < 0.001* probability levels, respectively.EC, plant growth regulator; PD, plant density.

**Supplementary Figure 5**


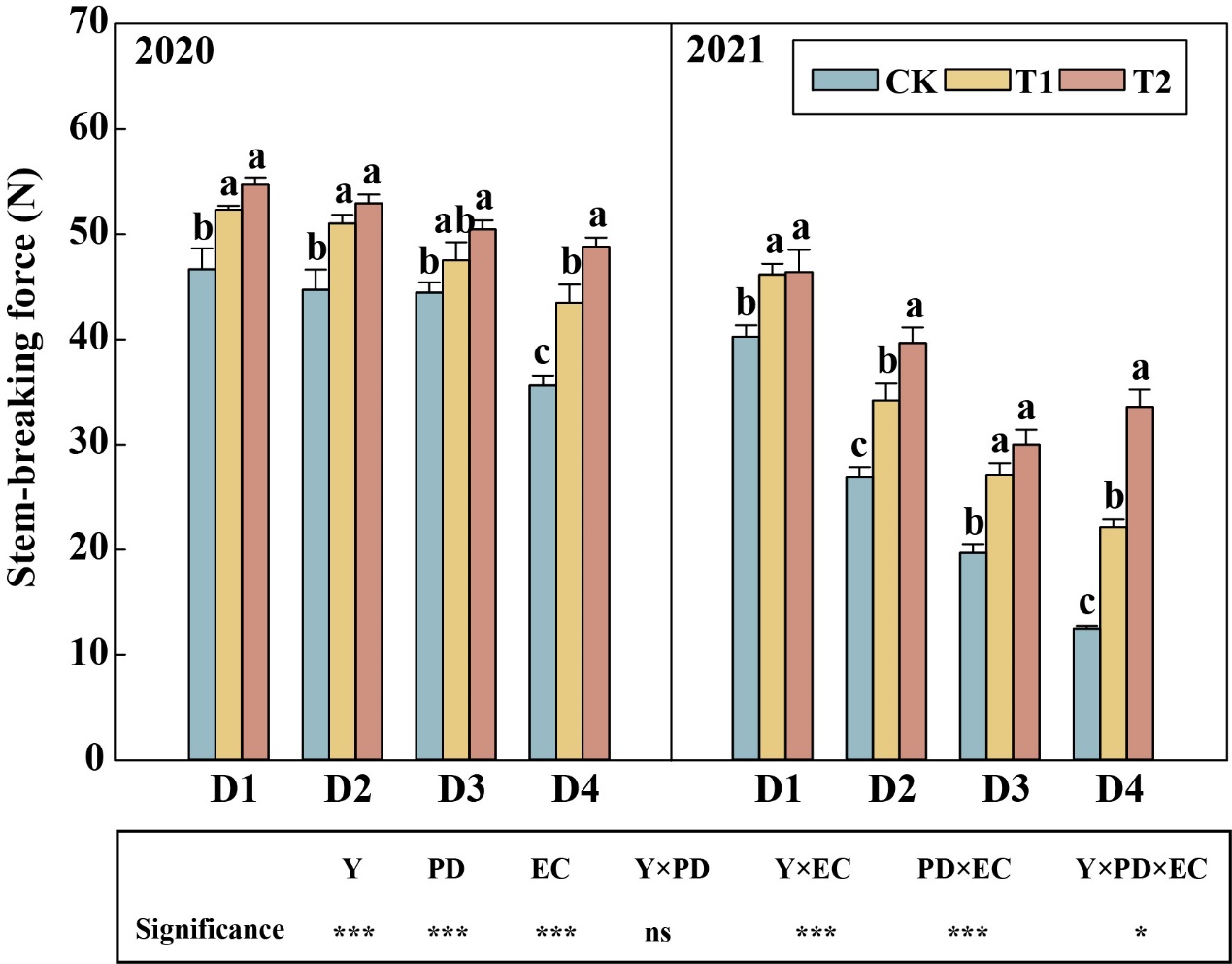


Effect of EC and PD on stem-breaking force 30 days after silking (30 DAS) for 2 years. And results of ANOVA on the effects of year (Y), plant density (PD), EC treatments (EC) and their interactions (PD×EC) on stem-breaking force in 2020–2021 are listed. Different lowercase letters indicate significant differences between EC treatments at each plant density. ns means non-significant. * and *** indicate significant differences at *P < 0.05 and < 0.001* probability levels, respectively. EC, plant growth regulator; PD, plant density.

**Supplementary Figure 6**


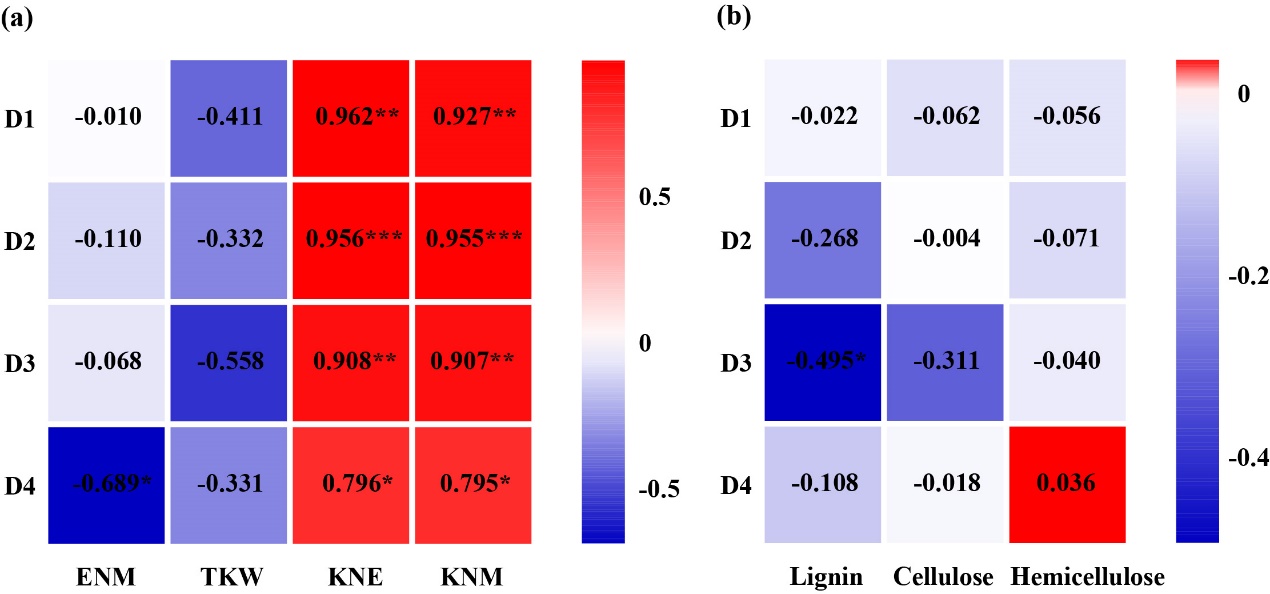


Relationships between ENM, TKW, KNE and KNM with yield under D1, D2, D3 and D4 in 2020–2021 (a). Relationships between lignin, cellulose and hemicellulose with KNE under D1, D2, D3 and D4 in 2021. “−” represents a negative correlation between the two indicators. *, **, and *** indicate significant differences at *P < 0*.*05*, *0*.*01*, and *0*.*001*, respectively. ENM, ear number per square meter; TKW, thousand kernel weight; KNE, kernel number per ear; KNM, kernel number per square meter.

**Supplementary Figure 7**


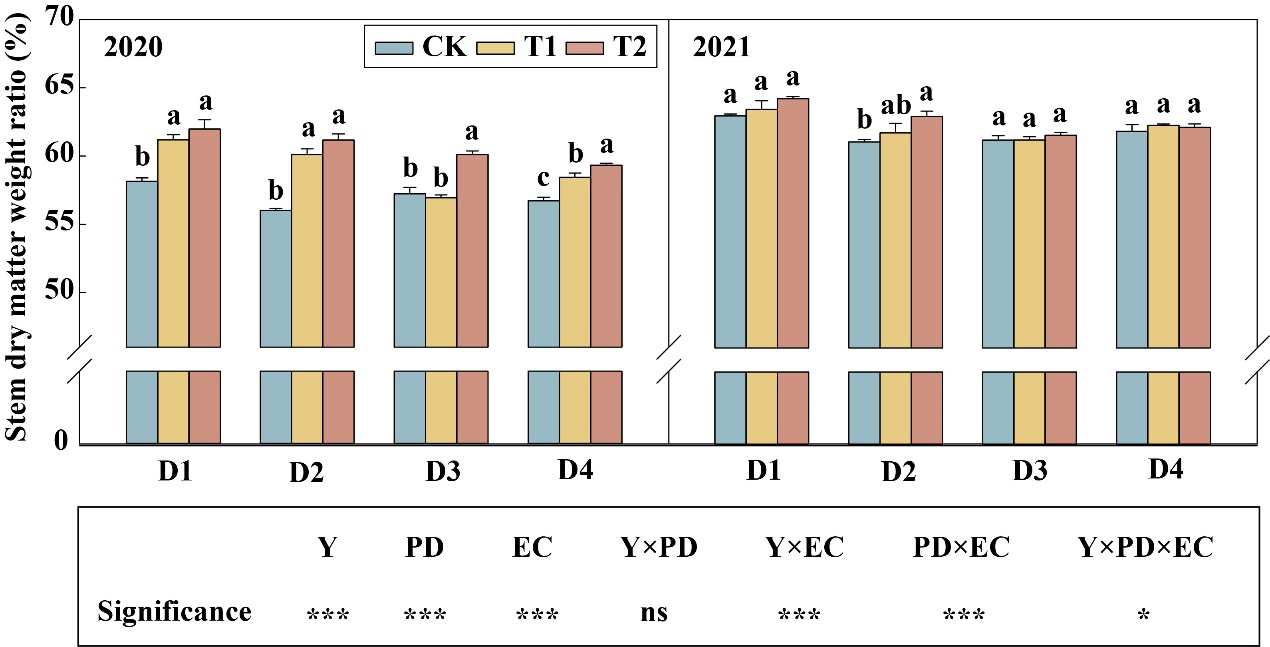


Effect of EC and PD on stem dry matter weight ratio at silking for 2 years. And results of ANOVA on the effects of year (Y), plant density (PD), EC treatments (EC) and their interactions (PD×EC) on stem-breaking force in 2020–2021 are listed. Different lowercase letters indicate significant differences between EC treatments at each plant density. ns means non-significant. * and *** indicate significant differences at *P < 0.05 and < 0.001* probability levels, respectively. EC, plant growth regulator; PD, plant density.
